# Effects of Vape Use on Oral Health: A Review of the Literature

**DOI:** 10.3390/medicina60030365

**Published:** 2024-02-21

**Authors:** Alin M. Iacob, Matías F. Escobedo Martínez, Enrique Barbeito Castro, Sonsoles Junquera Olay, Sonsoles Olay García, Luis Manuel Junquera Gutiérrez

**Affiliations:** 1 Asturian Institute of Dentistry, Oviedo University, 33003 Oviedo, Spain; alini63@yahoo.com; 2 Department of Integrated Adult Dentistry, School of Dentistry, University of Oviedo, 33003 Oviedo, Spain; olaymaria@uniovi.es; 3 Department of Gastroenterology, Hospital Universitario Central de Asturias, 33003 Oviedo, Spain; e.barbeitocastro@gmail.com; 4 Department of Radiology, Hospital Universitario Central de Asturias, 33003 Oviedo, Spain; junqueraolays@gmail.com; 5 Head Department of Oral and Maxillofacial Surgery and Oral Medicine, School of Dentistry, Oviedo University, 33003 Oviedo, Spain; junquera@uniovi.es

**Keywords:** oral health, electronic cigarettes (e-cigs), vaping, smoking, periodontitis, caries

## Abstract

*Background and Objectives*: The widespread use of tobacco has evolved with the popularity of vapes, especially among young people, despite the lack of clarity in warnings about their risks. Studies indicate the need for more effective communication about the oral risks of vaping. In addition to systemic, respiratory, and cardiovascular effects, vaping is associated with an increased risk of gingivitis and periodontal disease as well as reduced antioxidant capacity of saliva. The objectives of this narrative review are to summarize the existing information in the literature on the effects of vaping at the oral level and to bring together knowledge about the mechanism of action of vaping in oral tissues. *Materials and Methods*: In the present study, articles were searched in PubMed, Elsevier Scopus, and Web of Science using the keywords “*oral health*”, “*vaping*”, and “*vape*”. Studies published in the last 6 years that addressed the effects of oral vaping were selected, including comparisons among vape users, smokers, and non-smokers. Repeated articles, prior to 2017 and in languages other than English, were excluded. Two review authors (A.M.I and M.F.E.M) independently selected the papers based on titles and abstracts and conducted a full review of the remaining papers. In cases of disagreement, a third reviewer was used. *Results*: A total of 113 results were obtained, distributed as 16 from PubMed, 35 from Web of Science, and 62 from Elsevier Scopus. After removing duplicates, 67 articles were filtered by reviewing titles and abstracts, and finally, 22 articles were selected for comprehensive reading. Subsequently, eight of these articles were chosen for qualitative synthesis and are presented in standardized tables. The sample size of all included studies was composed of 31,647 participants, (14,477 male and 17,170 female) with a mean of 35.016 ± 7.57 years of age. *Conclusions:* This review indicates that the use of vapes is associated with an increased risk of periodontitis and caries. Although users experience more oral problems than non-smokers, these are less severe than those of traditional smokers. The widespread prevalence, especially among young people, highlights the urgency of awareness campaigns to warn of risks and understand potential harm.

## 1. Introduction

Tobacco has been a substance used in many social environments of society for centuries. Its consumption was so widespread that the possibility that it produced positive effects on health was raised. In addition, the use of electronic nicotine-releasing devices (ENRDs) has increased in recent years, especially among adolescents and young adults [1,2]. It has been observed that the current presentation of these devices and the available commercial alerts are not clear enough to make consumers aware that vaping produces harmful effects [3]. Studies like that by Hang et al. postulate that better communication is needed to disseminate the harmful potential of vaping in the oral environment and note the importance of dentists as entities to help disseminate the real information about these devices [2]. It has been observed that the majority of ENRD users are likely to receive instructions from dentists to stop using these devices [4].

The first identification of “vape” terminology dates back to the 1960s, when inventor H. Gilbert introduced the first alternative to the conventional cigarette [5]. This device represented an innovation for the cessation of the taboo habit (a practice widely spread at the time) and lacked nicotine. Over the course of history, this device was progressively modified until it reached the current form of vapes [6].

The question that arises is whether this device can effectively contribute to the suppression of smoking. The World Health Organization (WHO) maintains that vaping cannot be considered an effective tool for quitting tobacco use [7]. Other specialists, such as the UK’s National Health Service, argue that vaping may be partially useful as long as nicotine doses are gradually reduced [8].

The mechanism of the vape consists of a battery that generates an electric current, which activates a filament located in an atomizer. This ignited filament causes the e-liquid (the inhaled substance) to evaporate in the cartridge, generating an aerosol [9]. It is important to note that the vapors produced by vaping are not only made up of water as each inhalation introduces nanoparticles, volatile organic compounds, carbonyls, heavy metals, and nicotine into our bodies, forming an amalgam of compounds potentially harmful to health [10]. In addition to this group, substances, such as formaldehyde, acetaldehyde and acrolein, that are considered to be possible cancer inducers are incorporated into the aerosol during filament ignition [10].

Therefore, vaping persists as an element detrimental to health, despite the misinformation circulating in our society about the absence of negative effects. The journal “Pediatrics” notes in an article that vaping use has become an increasingly common and harmful trend among young people. In addition, it highlights that future efforts should examine the progression and toxicity of vape use among young people and educate them about the potential dangers of these behaviors [10].

Undoubtedly, its use can be double-edged. Although the possibility of quitting smoking through these devices was previously considered, recent studies suggest the probability of developing a new addiction, especially in individuals who have never used nicotine, and this is more evident in the younger population [9,10].

In the clinical setting of the oral cavity, there is ample evidence establishing a connection between vaping smoke and an increased risk of developing gingivitis and periodontal disease [11,12,13,14]. The use of the vape pen can introduce certain bacteria that contribute to the development of oral diseases in our body. In addition, there are studies that suggest that quitting vaping could be associated with an improvement in oral health [14]. In addition, the use of e-cigarettes has a negative impact on the antioxidant capacity of saliva, comparable to the effect observed in traditional cigarette smokers when compared to non-smokers. This decrease in antioxidant capacity translates into a decrease in the defensive capacity of the immune system in the oral cavity [15].

Therefore, given all the information provided, it is imperative to look for new data on vapers to give us a more complete and realistic perspective of the impact that these new trends can have on our health.

## 2. Objectives

The objective of this literature review is summarizing the information present in the literature on the effects of vaping at the oral level and bringing together knowledge about the mechanism of action of vapes at the level of oral tissues.

## 3. Materials and Methods

This narrative review has been carried out following a series of steps that are set out below.

*Search Strategy*

On 19 October 2023, a bibliographic search of three databases, PubMed, Elsevier Scopus, and Web of Science, was carried out, using a search strategy formulated by combining the keywords “*oral Health*” *[MeSH]*, “*vaping*”, and “*vape*” and the Boolean operators “OR” and “AND”, from which the following syntax resulted: ““*oral health*” *AND (*“*vaping*” *OR* “*vape*”)”.

*Inclusion Criteria*

Articles published in the last 6 years were selected. We included all articles in English that seemed relevant and could provide us with information about the effects of oral vaping and the possible etiology, epidemiology, and diagnosis of diseases of the mouth that are related to the administration of vapes. In addition, articles comparing patients who used vapes with those who smoked tobacco and non-smokers have been included.

*Exclusion Criteria*

We excluded all articles that did not study the consequences of oral vaping as well as those that only studied tobacco use, both systematic and narrative reviews, and letters to the editor.

*Study Selection and Data Collection*

Initially, the following were eliminated: (a) duplicate articles; (b) pre-2017 articles; (c) non-English language articles; (d) articles with patients < 18 years of age. Subsequently, independent reviewers (A.M.I. and E.B.C.) made the second selection through the reading of the titles and the abstracts of articles that potentially met the inclusion criteria. Finally, these works were read and analyzed in their entirety for their inclusion or exclusion in the literature review. In cases where there was no agreement for the inclusion or exclusion of an article, it was submitted to a third reviewer (M.F.E.M).

## 4. Results

**Figure 1:**
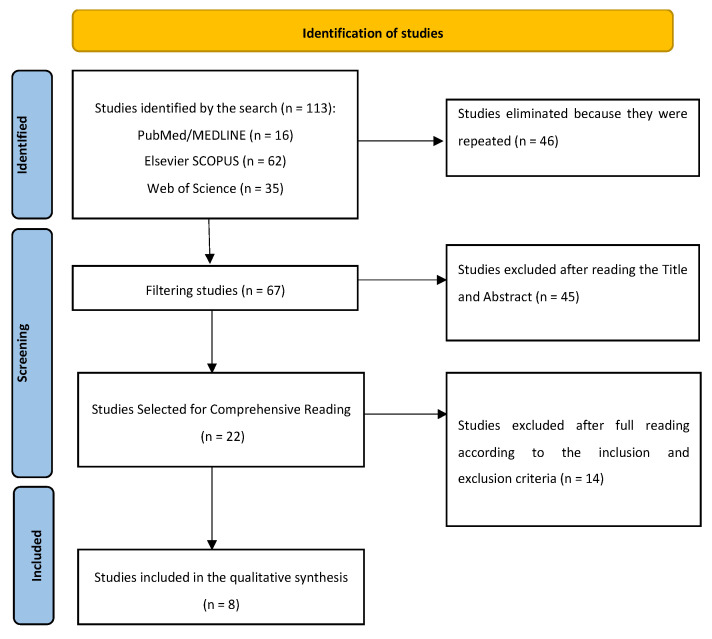
Flowchart explaining the series of steps that have been followed for the selection of articles included in the qualitative synthesis.

The syntax was entered into the different databases (Figure 1—see flowchart) and obtained a total of 113 results, of which 16 were obtained from the PubMed database, 35 from Web of Science, and 62 from Elsevier Scopus. Subsequently, duplicates were eliminated, resulting in 67 articles to be filtered by the reading of each title and abstract. After this step, a total of 22 articles were obtained for comprehensive reading.

Finally, after reading them completely, eight articles were selected for the qualitative synthesis, which are included in a standardized table (Table 1). The included articles were from the following countries: one in Saudi Arabia, one in Malaysia, four in the United States, one in South Korea, and one in Switzerland. Three of them were cohort studies, two case control studies, two in vitro studies, and one cross-sectional study. A table was created (Table 2) including the author and year of publication of the articles, the number of patients included in the studies, the gender of patients classified as male or female and number in each category, and the age of the participants expressed as mean age or age range depending on the case.

### Description of the Studies

The study carried out by Javed et al. [16] aimed to compare periodontal parameters and self-perceived oral symptoms among cigarette smokers, vaping individuals, and non-smokers. The research was conducted as a comparative study and received research support. The study focused on the medical field and investigated the potential impact of vaping on periodontal health. The study’s findings revealed that a large portion of patients reported quitting smoking combustible cigarettes as the most common reason for vaping. Some individuals reported vaping for social entertainment and fun. This suggests that vaping may serve as a smoking cessation aid for some individuals, while for others, it may be more of a recreational activity. In addition, the comparison of periodontal parameters and self-perceived oral symptoms among the different groups sheds light on the possible oral health implications of vaping, as the non-smokers in the study had the least bleeding on probing and the lowest plaque index on examination.

The case-control study conducted by Ghazali et al. [17] aimed to investigate the oral health of smokers and users of ENRDs. The study compared the oral health status of these two groups with a focus on identifying potential differences in oral health outcomes. The research design involved the selection of a group of smokers, a group of ENRD users as cases, and a matched group of non-smokers and non-ENRD users as controls. Several oral health parameters were evaluated and compared between the two groups, including periodontal health, dental caries, oral hygiene practices, and oral mucosal lesions. The study found that smokers exhibited a higher prevalence of periodontal disease compared to non-smokers. In addition, smokers were more likely to have tooth decay and poorer oral hygiene practices. On the other hand, ENRD users showed similar oral health outcomes to non-ENRD users in terms of periodontal health, tooth decay, and oral hygiene practices. However, the study also identified a possible association between the use of ENRDs and oral mucosal lesions, warranting further research into this aspect of oral health among e-cigarette users.

The study conducted by Ye et al. [18] investigated the levels of inflammatory biomarkers and growth factors in saliva and crevicular fluid (CF) of ENRD users, cigarette smokers, dual smokers, and non-users. The researchers found that ENRD users had significantly elevated levels of inflammatory biomarkers, such as interleukin-1β (IL-1β) and interferon-γ (IFN-γ), in their CF compared to non-users. In addition, ENRD users showed increased levels of growth factors, including the epidermal growth factor (EGF) and the vascular endothelial growth factor (VEGF), in their CF. These findings suggest that the use of ENRDs may have a remarkable impact on oral inflammatory response and tissue repair processes, as reflected in biomarker levels in CF.

The study by Jeong et al. [19] studied the associations between the use of ENRDs and conventional cigarettes and periodontal disease in South Korean adults. The research is particularly relevant given the increasing implementation of anti-smoking laws in South Korea. The authors stated that periodontal disease is much more prevalent in smokers of both conventional cigarettes and vapes than in non-smokers. However, both vaping and smoking were significantly linked to tooth decay and other dental disorders. The study suggested that vaping is not a safe alternative to tobacco and that smoking must be quit in any form for oral health to be maintained.

The study conducted by Vemulapalli et al. [20] investigated the association between vaping and untreated cavities. Using data from the National Health and Nutrition Examination Survey, the study employed an odds ratio analysis to assess the relationship between vaping and untreated cavities. The results indicated a significant association between vaping and untreated cavities, suggesting that people who vape are more likely to have untreated cavities. This finding has implications for dental health and underscores the importance of addressing vaping as a potential risk factor for oral health problems such as cavities.

Irusa et al. [21] compared the risk of caries between patients who use vapes or e-cigarettes and those who do not. The study used machine learning techniques to analyze the risk of tooth decay associated with vaping. The results of the study provide insight into the potential impact of vaping on tooth decay. This study contributes to understanding the oral health implications of the use of ENRDs and provides valuable information for dental and public health professionals.

Ramenzoni et al. [22] conducted an in vitro study to evaluate the cytotoxic and inflammatory effects of e-cigarettes and traditional cigarettes on oral gingival cells. The study aimed to compare ENRDs as a safer alternative to traditional cigarettes. The findings indicated that ENRDs are commonly marketed as a safer alternative to traditional cigarettes as well as a smoking substitution or cessation tool. This suggests that there is a perception that ENRDs are less harmful than traditional cigarettes, which may influence their usage patterns.

Xu et al. [23] examined the effects of e-liquids on biofilm formation and the growth of oral commensal streptococcal communities as well as the population of other opportunistic pathogens [22,23]. The study found that flavored e-liquids had a more detrimental effect on biofilm formation and the growth of oral commensal bacteria compared to unflavored e-liquids. This suggests that the flavoring agents in e-liquids may play a significant role in influencing oral microbiota.

Overall, it has been noted in the articles included in this review that vaping patients have some increased risk for dental sedation, so dentists should be aware of vaping patients [24,25]. On the other hand, it has been observed that the use of these devices increases the occurrence of complications in existing oral pathologies [26]. In addition, vaping must be considered by dentists as an important piece of information for the inclusion of a medical record [21] Therefore, dentists should remain alert to the emergence of new information in order to educate patients and warn them of the risks that this practice may have on their oral health [27].

## 5. Discussion

Vape use has increased considerably in recent years, leading to a growth in research into its potential impact on oral health. Several studies have investigated the effects of vapes on oral tissues, periodontal health, and the development of oral diseases [6,28]. These studies have shed light on various aspects of the relationship between the use of ENRDs and oral health, including the biological effects of ENRDs, the relevance to oral health, and the potential oral health consequences of their use in different populations. The literature has highlighted the need to understand the potential implications of the use of ENRDs for oral health, especially given the growing popularity of these devices as alternatives to traditional smoking [6]. It should be noted that the involvement of smoking in the progression of periodontal diseases as well as in other pathologies of the oral cavity, like oral cancer, has been well documented, emphasizing the importance of exploring the impact of vapes on oral health [29]. On the other hand, the growing popularity of pipe tobacco and the use of ENRDs has raised implications for oral care, demanding a deeper understanding of the biological effects of these products on oral health [30].

Other authors, such as Almeida et al., have explored the effects of aerosols from these devices on the cells and tissues of the oral cavity, providing information on possible relapses into traditional smoking due to vaping [6]. In addition, the growing number of studies conducted to explore the effects of ENRDs on oral health underscores the need for comprehensive research in this area [14]. As the popularity of vaping continues to rise, it is vital that dental professionals stay informed and provide patients with accurate information based on emerging data [31]. On the other hand, research has delved into the self-reported adverse effects of the use of ENRDs among dental students, highlighting the importance of including information on the oral/general health impact of these in the curricula of dental and health disciplines [27]. However, a cross-sectional questionnaire study conducted by Huilgol et al. has underscored the need for more research on the association between the use of these devices and oral health, especially in adolescents [32].

Studies have observed damage to cell DNA, increased oxidative stress, inflammation, and alterations in healing [33]. The impact of these on DNA damage in human oral cells, changes in antioxidant capacity and nucleotide metabolites in saliva, and the antibacterial properties of saliva has also been the subject of research, calling for further longitudinal studies to assess the effect of these changes on oral health [15,29]. On the other hand, the evaluation of oral changes resulting from the use of ENRDs and the review of the literature related to traditional cigarettes and vapes and oral health have provided valuable information on the potential oral health consequences of their use [34,35].

Based on the references included in our qualitative synthesis, it can be concluded that vaping is associated with several adverse effects on oral health. Xu et al. [23] demonstrated that flavored vaping liquids have a more detrimental effect on biofilm formation and the growth of oral commensal bacteria compared to unflavored liquids. This suggests that additives used to flavor vaping liquids may exacerbate oral health problems. In addition, Jeong et al. [19] found that both the use of ENRDs and conventional are significantly associated with increased rates of periodontal disease, indicating that regardless of the type of cigarette used, there is a negative impact on periodontal health. Notably, Ye et al. [18] reported the presence of inflammatory biomarkers and growth factors in the saliva and gingival crevicular fluid of ENRD users, cigarette smokers, and dual smokers. This statement suggests that the use of ENRDs contributes to an inflammatory response in the oral cavity, which may have implications for overall oral health. Javed et al. [16] compared periodontal parameters and self-perceived oral symptoms among cigarette smokers, vaping individuals, and non-smokers and found differences indicating possible oral health risks associated with vaping. At the same time, it has been observed that patients who smoke conventional cigarettes have more gingival pain and other oral symptoms than those who vape [16]. Ramenzoni et al. [22] also demonstrated the cytotoxic and inflammatory effects of ENRDs on oral gingival cells, further emphasizing the potential damage caused by them in oral tissues. On the other hand, Irusa et al. [21] compared the risk of tooth decay between patients who vape and those who do not, indicating a possible association between vaping and an increased risk of tooth decay. Finally, Vemulapalli et al. [20] investigated the association between appraisal and untreated caries, evidencing the need to explore the oral health implications of vaping in the context of dental caries.

According to Bardellini et al., hyperplastic candidiasis is identified as a common oral mucosal lesion among e-cigarette users. The study found a high prevalence of hyperplastic candidiasis in the retrocomissural area of individuals who use electronic cigarettes, with a prevalence rate of 17.8%. The authors suggest that this high prevalence may be attributed to a pH alteration induced by the chemical constituents present in e-liquids. This finding underscores the potential impact of e-cigarette usage on the oral mucosa, particularly in relation to the development of hyperplastic candidiasis in specific oral regions [36]. This is particularly concerning as the aerosol produced by e-cigarettes can contain harmful ingredients, including flavorants and solvents, in addition to nicotine [37]. Further investigation regarding vaping effects on oral mucosa pathology and morphology need to be undertaken.

The ethical considerations of vaping involve public health, harm reduction, messaging, and regulations. Concerns have arisen regarding the pro-vaping messages dominating social media, raising ethical questions about the industry’s impact on public perception and behavior [38]. While e-cigarettes may serve as a tool for risk reduction among smokers, there are concerns regarding the uptake of vaping among youth and never smokers, necessitating a balanced approach to address potential public health implications [39]. Banning non-therapeutic nicotine vaping products presents an ethical dilemma, as it may deny addicted smokers access to a potentially less harmful alternative for quitting smoking [40]. It is imperative to gain a better understanding of vaping outcomes and adolescents’ perceptions while identifying potential ways to lessen or eradicate the health burdens associated with vaping [41]. The ethical dilemma surrounding restricting access to vaping products is complex, and common-sense reasons for prioritizing the young do not necessarily apply to vaping restrictions and harm reduction [42].

The principal limitation of this work was the scarcity of quantitative studies assessing vaping effects on oral health not only regarding periodontal health but also in the field of oral pathology. The lack of homogeneity among the studies was also an important factor that limited our capability to extract consistent conclusions on the appraised aspects. Finally, our review was also narrowed by the small number of studies comparing exclusive vape consumers to healthy subjects. We deduce that exclusive vape users and healthy subjects should be assessed quantitatively in the future to draw more consistent conclusions in this area; fields such as oral pathology should also be included in these types of studies as they represent some prevalent conditions that are being diagnosed daily by dental professionals.

## 6. Conclusions

From the present study, we can conclude that vaping may be linked to increased rates of periodontitis as well as an increased risk of developing a carious pathology. The literature suggests that patients who vape could experience more gum pain and oral symptoms than non-smokers, although this would be less than those who smoke conventional cigarettes. Also, the widespread use of vapes, especially among young people, may require the carrying out of more effective awareness campaigns to warn of their risks for increasing the awareness of the population of the possible potential harms caused by their use in the oral cavity.

## Figures and Tables

**Table 1:** Studies collected for qualitative synthesis.

Number	Author (Year of Publication)	Full Title	Summary and Conclusions
1	Javed et al. (2017) [16]	Comparison of periodontal parameters and self-perceived oral symptoms among cigarettes smokers, individuals vaping electronic cigarettes, and never-smokers	The study compared periodontal parameters and self-perceived oral symptoms between cigarettes smokers, individuals using e-cigarettes, and non-smokers. The study concluded that the periodontal inflammation and self-perceived oral symptoms were poorer among cigarette smokers than among vaping individuals.
2	Ghazali et al. (2018) [17]	Oral health of smokers and e-cigarette users: A case-control study	The study compares the oral health of cigarette, e-cigarette, and vape smokers and non-smokers. The study concluded that e-cigarettes have potentially detrimental effects on oral health.
3	Ye et al. (2020) [18]	Inflammatory biomarkers and growth factors in saliva and gingival crevicular fluid of e-cigarette users, cigarette smokers, and dual smokers: A pilot study	The study was on inflammatory biomarkers and growth factors in saliva and crevicular fluid of e-cigarette users, cigarette smokers, and both. In conclusion, smoking/vaping produces significant effects on oral health.
4	Jeong et al. (2020) [19]	Associations of electronic and conventional cigarette use with periodontal disease in South Korean adults	The study examines the associations of e-cigarette and conventional cigarette use with periodontal disease in South Korean adults. They concluded that smoking and vaping produce incremented rates of periodontal disease.
5	Velmulapalli et al. (2021) [20]	Association between vaping and untreated caries A cross-sectional study of National Health and Nutrition Examination Survey 2017–2018 data	The study examines the relationship between vaping and the presence of untreated cavities. In conclusion, both vaping and dual smoking are associated with an increased occurrence of untreated caries.
6	Irusa et al. (2022) [21]	A comparison of the caries risk between patients who use vapes or electronic cigarettes and those who do not: A cross-sectional study	The study deals with the risk of tooth decay associated with vaping. The authors concluded that vaping patients had a higher risk of developing caries.
7	Ramenzoni et al. (2022) [22]	Cytotoxic and inflammatory effects of electronic and traditional cigarettes on oral gingival cells using a novel automated smoking instrument: An in vitro study	The study investigated the effects of e-cigarettes and traditional cigarettes on oral gingival cells using a novel automated smoking instrument. The conclusions drawn stated that e-cig smoking may contribute to the cell damage of oral tissue and tissue inflammation.
8	Xu et al. (2022) [23]	Mechanistic effects of e-liquids on biofilm formation and growth of oral commensal streptococcal communities: Effect of flavoring agents	The mechanistic effects of e-liquids on biofilm formation and the growth of oral commensal streptococcal communities were investigated. The findings of the study indicate that flavored e-liquids have a more detrimental impact on the formation and growth of oral commensal bacteria compared to unflavored e-liquids.

**Table 2:** Demographic data of the included articles.

Number	Author (Year of Publication)	Number of Patients	Gender (M: Male//F: Female) (Number of Cases)	Age (Mean Age/Age Range)
1	Javed et al. (2017) [16]	94	M (94)	Mean age: 39.87 ± 2.17 years
2	Ghazali et al. (2018) [17]	120	M (89)//F (31)	Mean age: 27.66 ± 7.58
3	Ye et al. (2020) [18]	48	M (24)//F (24)	Mean age: 37.56 ± 13.03 years
4	Jeong et al. (2020) [19]	13,551	M (5715)//F (7836)	Mean age: 29.18 ± 4.24 years
5	Velmulapalli et al. (2021) [20]	4618	M (2234)//F (2384)	Mean age: 41.81 ± 6.11 years
6	Irusa et al. (2022) [21]	13,216	M (6321)//F (6895)	Age range Age: 16–25 years—845 patients Age: 26–40 years—4336 patients Age: >40 years—8014 patients
7	Ramenzoni et al. (2022) [22]	No data	No data	No data
8	Xu et al. (2022) [23]	No data	No data	No data

No data: no information.
